# IL-33/ST2 immune responses to respiratory bacteria in pediatric asthma

**DOI:** 10.1038/srep43426

**Published:** 2017-03-06

**Authors:** Isabell Hentschke, Anna Graser, Volker O. Melichar, Alexander Kiefer, Theodor Zimmermann, Bettina Kroß, Patricia Haag, Paraskevi Xepapadaki, Nikolaos G. Papadopoulos, Christian Bogdan, Susetta Finotto

**Affiliations:** 1 Abteilung für Molekulare Pneumologie, Friedrich-Alexander-Universität (FAU) Erlangen-Nürnberg, Universitätsklinikum Erlangen, Erlangen, Germany; 2 Kinder- und Jugendklinik, Pädiatrische Pneumologie, Friedrich-Alexander-Universität (FAU) Erlangen-Nürnberg, Universitätsklinikum Erlangen, Erlangen, Germany; 3 Allergy and Clinical Immunology Unit, 2nd Pediatric Clinic, National and Kapodistrian University of Athens, Athens, Greece; 4 Division of Infection, Inflammation and Respiratory Medicine, The University of Manchester, Manchester M13 9PL, UK; 5 Mikrobiologisches Institut – Klinische Mikrobiologie, Immunologie und Hygiene, Friedrich-Alexander-Universität (FAU) Erlangen-Nürnberg, Universitätsklinikum Erlangen, Erlangen, Germany

## Abstract

Here we investigated the relationship between local bacterial colonization and anti-bacterial immune responses in pre-school asthmatic and control children within the EU-wide study PreDicta. In this cohort of pre-school asthmatic children, nasopharyngeal colonization with Gram-negative bacteria such as *Haemophilus influenzae* and *Moraxella catarrhalis* was found to be associated with the highest interferon beta (IFNβ) and IL-33 levels in the nasal pharyngeal fluids (NPF). IL33R-ST2 was found induced in the blood of asthmatic children with additional Gram + bacteria in the nasopharynx (Gr+/−). Furthermore, asthmatic children had more episodes of infection that required antibiotic therapy than the control group. Treatment with antibiotics associated with reduced ST2 in blood cells of both asthmatic and control children and reduced IL-33 levels in the airways of asthmatic children. In the absence of *Staphylococcus (S.) aureus* in NPF, antibiotic therapy associated with decreased IL-33 levels in the NPF and lower ST2 values in the blood of control children but not of asthmatic children. These data suggest that, in asthmatic children, Gram- bacteria, which persist after antibiotic therapy, contributes to IL-33 locally and associated with Gr + bacteria colonization in the airways, inhibited IFN-β and in the absence *of Staphylococcus (S.) aureus,* induced ST2 bearing cells in their blood.

Bacterial infections are known to trigger asthma exacerbations^1^,^2^,^3^. It is also known that recurrent viral and bacterial infections during childhood can promote the susceptibility to allergy and asthma^4^. The microbial milieu has been shown to influence the priming of the immune system already in utero as well as in early childhood^5^,^6^,^7^,^8^. However, we do not know yet which immune responses are present in combination with distinct bacteria colonization in pediatric asthma.

Interferon beta (IFNβ) is a protein that is induced by both viral and non-viral pathogens and best known for its strong antiviral, antibacterial and immunoregulatory effects^9^,^10^,^11^. IL-33 is a cytokine known to be released by damaged epithelial cells where infectious agents and/or allergens can spread through blood and into tissues. It is a cytokine of the innate immune response and activates innate lymphoid cells type 2 (ILC2) and Th2 cells to produce IL-5 and IL-13 after binding to its receptor suppression of tumorigenicity (ST2), also termed IL-33 receptor (IL-33R). These Th2 cytokines were found to be increased in the nasopharynx of asthmatic patients^12^. Moreover, recently IFN-β has been shown to inhibit ILC2 cells which carry the type I IFN-R^13^.

The relationship between the bacterial colonization and the pathogenesis of allergic asthma has become a field of intense research^2^,^14^,^15^. Recently, it has been found that the lower respiratory tract, which previously was assumed to be sterile, is colonized by several microorganisms^16^. The microbiota in the airways of asthmatic subjects showed a higher bacterial diversity compared with the one of healthy subjects^16^,^17^.

At the moment there is increasing evidence that the colonization of the airways with microorganisms can trigger the onset and the development of asthma, but can also have protective effects. In this study we analyzed two cohorts of pre-school children one with and the second without asthma recruited within the Europe-wide study PreDicta (Post-infectious immune reprogramming and its association with persistence and chronicity of respiratory allergic diseases) and addressed the question whether the presence of distinct bacteria in their nasal pharyngeal fluids (NPF) as well as previous antibiotic therapy were associated with different antibacterial immune responses such as IL-33 and IFNβ production in their NPF and IL-33R/ST2 in the blood.

## Results

### Differences in bacterial nasopharyngeal colonization of pre-school children with asthma compared to healthy children

The demographic and clinical data of the pre-school children analyzed in this study are reported in Table 1. To investigate the relationship between bacterial nasopharyngeal colonization and the immune responses, we first looked for the common bacterial colonization of the nasopharynx, as well as for Gram positive and Gram negative bacteria, which might cause airway infections, especially in pre-school age in two cohorts of pre-school children with and without asthma.

**Figure 1:**
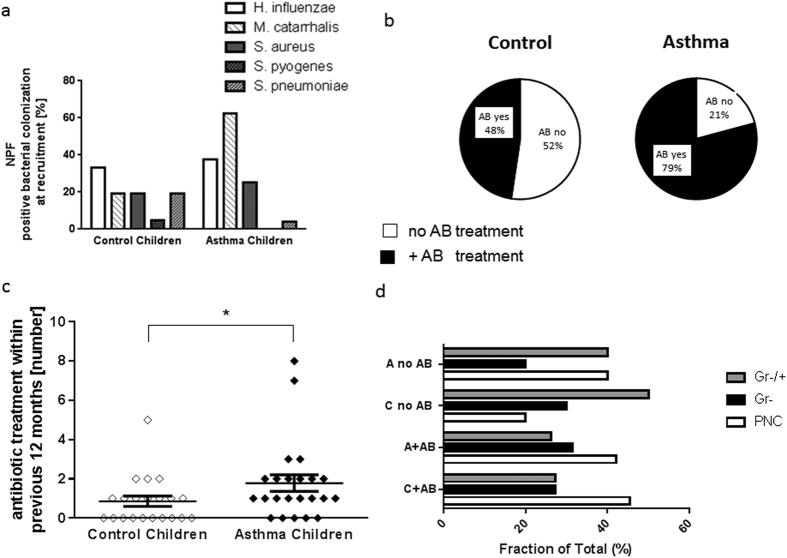
More frequent antibiotic treatment in children with asthma (A) within 12 months before recruitment. (**a**) Percentage of colonization of the five main bacteria *Haemophilus influenza (H. influenzae*: Gram-), *Moraxella catarrhalis (M. catarrhalis*: Gram-), *Staphylococcus aureus (S. aureus:* Gram+), *Streptococcus pyogenes (S. pyogenes*: Gram+) and *pneumoniae (S. pneumonia:* Gram+). NPF: Nasalpharyngeal fluid. (**b,c**). More frequent antibiotic treatment in children with asthma (A) within 12 months before recruitment (**d**) Higher percentage of colonization with diverse Gram positive and Gram negative bacteria in children without antibiotic therapy. (**a**: Control (C): n = 21; Asthma (A): n = 24 ; **b**: C: n = 10,11; A: n = 19, 5 ; **c**: n = 21, 24, p = 0,037; **d**: beginning from the top A no AB: n = 2,1,2,8,3,2,5,6,8,3,3,5).

Here we found a prevalence of five facultative pathogen bacteria that are known to be associated with asthma development (Fig. 1a).

In order to start analysing the influence of different bacterial colonization on asthma, we subdivided both cohorts in accordance to their bacterial nasopharyngeal colonization: Children that had saprophytic germs and bacteria that are physiological in the nasopharyngeal microbiome (PNC), or children that had additional or exclusively Gram negative bacteria in their nasopharyngeal fluid (Gram−). The Gram negative respiratory bacteria for which the nasopharyngeal fluid was analysed are *Haemophilus influenzae* and *Moraxella catarrhalis* with or without physiological flora. The third subgroup consists of children who have additional to physiological and/or Gram negative bacterial colonization also special Gram positive bacteria in their nasopharynx (Gram−/+). These Gram positive bacteria are *Staphylococcus aureus* and *Streptococcus* species.

As shown in Fig. 1a, the percentage of special bacterial colonization was increased in the asthma group as compared to the control group at the recruitment (B0). *Moraxella catarrhalis* was detected more frequently in asthmatics as compared to control children.

### Increased frequency of antibiotic therapy in asthmatic children as compared to healthy controls

As antibiotic treatment has a profound influence on the bacterial flora, we first established for every child the number of antibiotic courses received during the past twelve months before recruitment (Table 2 and Fig. 1b).

While half of the control children did not receive antibiotic treatment during infections in the previous twelve months, only 21% of children with asthma were not treated with an antibiotic therapy (Fig. 1b). As a result, asthmatic children received more often antibiotic therapy than control children (Fig. 1c). The more striking increase in antibiotic treatment was observed in asthmatic children with *S. aureus* detected in their NPF (3.3 fold induction) as compared to the control group (Table 2). Moreover, 84% of the asthmatic children with antibiotic treatment were also under steroid therapy (Table 3). Finally, we analyzed the fraction of children with different bacteria flora without antibiotic treatment (no AB) and with antibiotic treatment (+AB) (Fig. 1d). Here we observed a reducing effect of antibiotic on the frequency of both asthmatic and control children with a mixed bacterial Gram positive and Gram negative colonization in NPF. Moreover, antibiotics did not decrease the percentage of children with Gram negative bacterial colonization in the NPF. Table 4 shows antibiotic treatment of control children at recruitment and Table 5 contains the relevant bacterial and medication data of the study children at the end of the study course (F4: 24 month follow-up visit).

### Higher IFNβ production in nasopharyngeal fluid of children with asthma and a Gram negative (Gr-) bacterial colonization in the nasopharynx

**Figure 2:**
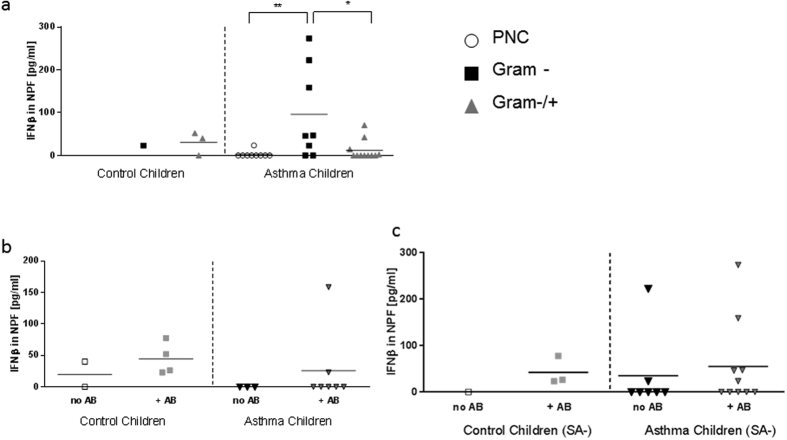
Gram negative germs are associated with higher levels of IFNβ in NPF of asthmatic children. (**a**) Increased IFNβ production in asthmatic children with a Gram negative bacterial colonization in nasopharyngeal fluid during the study (p = 0.0095, p = 0.0101; baseline and follow up visits). (**b**) Treatment with antibiotics ( + AB) or (**c**) in absence of *S. aureus* (SA-) had no significant effect on IFNβ in NPF of control and asthmatic children. (**a:** n = 1,3,9,8,11; **b**: n = 2,4,3,7; **c**: n = 1,3,7,10). Statistic values: (**a**) Control children (C) Gram+/− vs Asthma children (A) Gram+/−: p = 0.12; (**b**) C no AB vs C + AB: p = 0.16; C no AB vs A no AB: p = 0.13; C + AB vs A + AB: p = 0.28; A no AB vs A + AB: p = 0.24; (**c**) C + AB-SA vs A + AB-SA: p = 0.41; A no AB-SA vs A + AB-SA: p = 0.33.

To understand the influence of local nasopharyngeal bacterial colonization on IFNβ production, we analyzed the production of IFNβ in the NPF of the study children (baseline and follow up visits). Here, we found significantly higher amounts of IFNβ in the NPF of asthmatic children with Gram negative nasopharyngeal colonization as compared to asthmatic children with physiological bacterial flora (physiological nasalpharyngeal colonization: PNC; p = 0.0095) or additional Gram-positive bacteria in the nasopharynx (Gram−/+; p = 0.0101; Fig. 2a). IFNβ levels in NPF were not affected by previous antibiotic treatment (Fig. 2b). Finally, the absence of *S. aureus* (SA-) in the NPF did not influence IFN-β release (Fig. 2c).

### Higher IL-33 production in nasopharyngeal fluid of children with asthma and a Gram negative (Gram-) bacterial colonization in the nasopharynx

**Figure 3:**
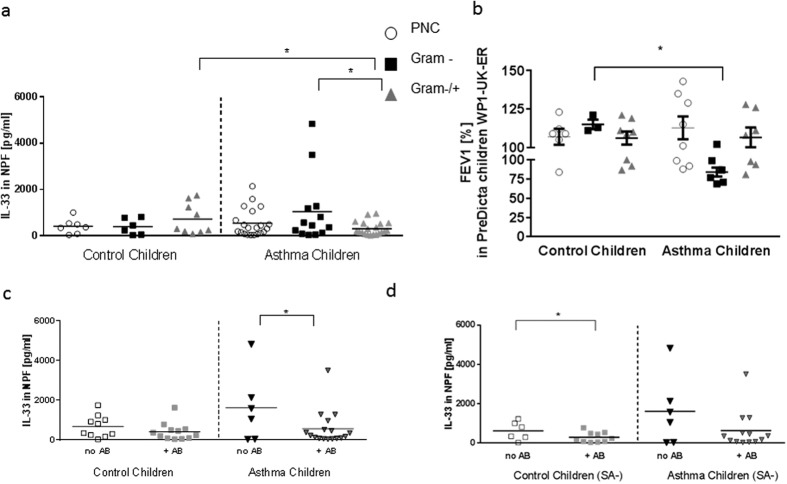
Increased IL-33 production in nasopharyngeal fluid of children with Asthma and Gram negative bacterial colonization in NPF. Decreased IL-33 production in children with Asthma after antibiotic therapy and in control children after antibiotic therapy in absence of *S. aureus* (**a**) Increased IL-33 production in the NPF of asthmatic children with Gram negative bacterial colonization at baseline and follow up visit as compared to asthmatic children with Gram negative and positive bacterial colonization (Gram−/+) (p = 0.0181). IL-33 levels in asthmatic children Gram−/+were significantly decreased as compared to the corresponding control group (p = 0.0121). (**b**) Lower values of FEV1 in children with asthma are associated with Gram negative nasopharyngeal bacterial colonization at time of recruitment (B0). Significantly lower percentage of FEV1 in children with asthma and *Haemophilus influenzae* or *Moraxella catarrhalis* than in healthy children (n = 4–8; p = 0,0238, Mann-Whitney U test). (**c**) Decreased IL-33 production measured in the NPF of asthmatic children after antibiotic therapy (p = 0.0274). (**d**) Decreased level of IL-33 measured in the NPF of control children in absence of *S. aureus* (SA-) in NPF after antibiotic treatment ( + AB) p = 0.045. (**a**: n = 7,6,9,21,13,20; **b**: n = 11,11,7,16; **c**: n = 6,10,6,13). Statistic values: (**a**) Control children (C) Gram+/− vs Asthma children (A) Gram+/−: p = 0.0121; C Gram- vs A Gram-: p = 0.15; C PNC vs A PNC: p = 0.29; C PNC vs C Gram-: p = 0.46; C PNC vs C Gram−/+: p = 0.14; C Gram- vs C Gram−/+: p = 0.14; A PNC vs A Gram-: p = 0.086; A PNC vs A Gram−/+: p = 0.058. (**c**) C no AB vs C + AB: p = 0.13 ; C + AB vs A + AB: p = 0.29; (**d**) C + AB-SA vs A + AB-SA: p = 0.14; A no AB-SA vs A + AB-SA: p = 0.067; C no AB-SA vs A no AB-SA: p = 0.10.

IL-33 is known to be produced by damaged epithelial cells and to shift the immune response towards the Th2 cytokines IL-5 and IL-13^18^. To test whether there is also a correlation between IL-33 production and bacterial colonization, we evaluated the IL-33 production in the NPF of the study children. By analyzing the NPF at different study visits (baseline and follow up visit) with regard to bacterial colonization, we could observe an increase in IL-33 production in the NPF of asthmatic children with a Gram- bacterial colonization as compared to children with additional Gram positive bacteria in the nasopharynx (Gram−/+; Fig. 3a, p = 0.0181). The asthmatic children with additional Gram + bacteria in the nasopharynx had also a significant reduction of IL-33 in their airways as compared to the correspondent control group of children (Fig. 3a, p = 0.0121).

### The presence of Gram negative bacteria in the nasopharyngeal tract is associated with lower FEV1 in children with asthma at the time of recruitment into the study

Since IL-33 is associated with asthma exacerbations^19^, we next looked at the forced expiratory volume of one second (FEV1). Here we found that children with asthma and Gram negative bacterial nasopharyngeal colonization had less relative FEV1 as compared to the healthy controls (Fig. 3b, p = 0.0238, n = 4–8). In conclusions, we observed that colonization with Gram negative respiratory pathogens is associated with an increased airway hyperresponsiveness in our cohort of asthmatic children (Fig. 3b) and induced IL-33 in the airways.

### Therapy with antibiotics is associated with decreased IL-33 in asthmatic children and in control children in the absence of *S. aureus* in nasopharyngeal fluid

We next reasoned that antibiotic therapy could influence IL-33 expression and release. We thus analyzed the baseline and follow-up data with regard to the antibiotic therapy. Here we could observe that only within the asthmatic group, antibiotic treatment was associated with reduced IL-33 levels in the NPF (Fig. 3c, p = 0.0274).

*S. aureus* is a common human pathogen which can cause severe infections in various tissues including the respiratory tract. Especially, some of the exotoxins (superantigens) produced by *S. aureus* are thought to be involved in the modulation and aggravation of airway inflammation^20^,^21^. We thus analyzed antibiotic therapy along with the absence of *S. aureus* and IL-33 production in NPF. We found that antibiotic therapy was associated with a significant reduction of IL-33 production only in control children that did not carry *S. aureus* in their NPF (Fig. 3d, p = 0.045).

### Increased *ST2* mRNA expression in the blood cells of asthmatic children with a Gram positive and negative (Gram+/−) bacterial colonization in the nasopharynx

**Figure 4:**
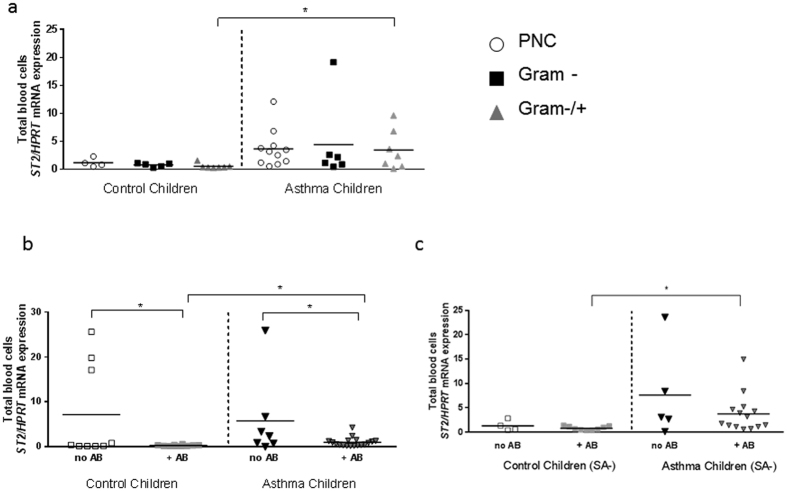
Higher levels of ST2 mRNA expression in asthmatic children with Gram positive and negative bacterial colonization in their airways. Decreased *ST2* mRNA expression in the blood of control children and asthmatic children after antibiotic treatment. (**a**) Higher Level of *ST2* mRNA expression in the blood of asthmatic children and diverse Gram positive and Gram negative bacterial nasopharyngeal colonization at baseline visit and follow-up visit (p = 0.027). (**b**) Decreased *ST2* mRNA measured in the blood of control (p = 0.027) and asthmatic (p = 0.018) children who were treated with antibiotics (+AB) compared to not treated (no AB) children. (**c**) Higher *ST2* mRNA expression in asthmatic children with antibiotic therapy independently of *S. aureus (SA)* (SA-: p = 0.018, SA included see **b**: p = 0.0174). (**a**: n = 4,5,10,12,6,7; **b**: n = 9,10,7,18; **c**: n = 4,9,6,14). Statistic values: (**a**) C Gram- vs A Gram-: p = 0.08; C PNC vs A PNC: p = 0.087; C PNC vs C Gram-: p = 0.17; C PNC vs C Gram−/+: p = 0.06; C Gram- vs C Gram−/+: p = 0.18; A PNC vs A Gram-: p = 0.38; A PNC vs A Gram−/+: p = 0.45; A Gram-vs A Gram−/+: p = 0.38 (**b**) C no AB vs C + AB: p = 0.026; C + AB vs A + AB: p = 0.0174; (**c**) C + AB-SA vs A + AB-SA: p = 0.0177; A no AB-SA vs A + AB-SA: p = 0.10; C no AB-SA vs A no AB-SA: p = 0.12.

As IFN-beta was found to be upregulated in the NPF of asthmatic children with Gram-negative colonization as compared to those children with asthma and a Gram−/+ bacteria colonization and has recently been shown to directly blunt IL-33 induced proliferation of bone marrow-derived ILC2 cells^13^, we next analyzed the expression of IL-33 receptor (R) (ST2: suppressor of tumorigenicity 2), signature of ILC2 cells and Th2 cells, in the peripheral blood of the cohorts of pre-school children taking into consideration their bacterial colonization in the NPF. Here we found that, the group of asthmatic children with a Gram negative and positive colonization (Gram−/+) in their airways, despite of the fact that they had lower IL-33 levels as compared to other subgroups of asthmatic children with different nasal-pharyngeal bacteria colonization (Fig. 3a), had a higher expression of *ST2* mRNA in the peripheral blood as compared to the corresponding subgroup of control children (Fig. 4a, p = 0.027). One possible explanation for this finding, to be further investigated in the future by expanding the number of children analyzed, could be the reduced IFNβ in the airways of these children (Fig. 2a) or other not yet identified factors regulating ST2 positive cells. Although the number of samples analyzed were limited, these children with sole Gram negative colonization of *H.influenzae* and/or *M.catarrhalis* had the highest IFNβ among the children analyzed (Fig. 2a) but also high IL-33 levels thus, as result, *ST2* mRNA in this group was not significantly regulated. In the systemic circulation, *ST2* mRNA expression is significantly higher in children with asthma than in their corresponding controls, so that the nasopharyngeal colonization might not predominantly influence systemic mRNA expression. Yet, antibiotic therapy might provide a target to reduce inflammation. Consequently, the influence of antibiotic therapy as it effects blood circulation was examined next to find a target to reduce inflammatory pathways.

### Therapy with antibiotics is associated with decreased ST2 in control and asthmatic children

We next analyzed the children in accordance to antibiotics treatment and then looked at *ST2* mRNA expression in the blood. Here, we found that *ST2* mRNA was down-regulated in the blood of control children, as well as asthmatic children treated with antibiotic as compared to the corresponding group without antibiotic treatment (no AB). Moreover, asthmatic children treated with antibiotics had higher levels of *ST2* mRNA compared to the corresponding control children treated with antibiotics (Fig. 4b, p = 0.0174).

### After antibiotic treatment, in the absence of *S. aureus* colonization in nasopharyngeal fluid, asthmatic children had higher values of *ST2* mRNA as compared to control children

To analyze the effect of *S. aureus* colonization on *ST2* mRNA we analyzed the antibiotic treatment effect without *S. aureus.* Here we found *ST2* mRNA upregulated in the blood of asthmatic children treated with antibiotics in the absence of *S. aureus* in their NPF compared to the corresponding control children subgroup (Fig. 4c, p = 0.018). Together these data suggest an inhibitory function of the antibiotic therapy on the IL-33/ST2 pathway in the absence of *S. aureus* in control children but not asthmatic children.

## Discussion

In this study we analyzed the immune responses of pre-school children with and without asthma taking into consideration their bacteria colonization in the NPF. Because the bacteria detected in the airways of these children are affected by the use of antibiotics during infections, we first investigated the use of antibiotics 12 months before the point of blood and NPF withdrawal. Not surprisingly, we noticed an increased use of antibiotics in asthmatic children as compared to control children. The most striking increase in antibiotic treatment was observed in asthmatic children with *S. aureus* detected in their NPF (3.3 fold induction) as compared to the control group. It is known that *S. aureus* can produce exotoxins with super-antigenic properties which represent a serious risk for human health^22^,^23^. These super-antigens are able to stimulate T cells and antigen-presenting cells already at very low concentrations and thus initiate a cascade of pro-inflammatory cytokines. It has been suggested that *S. aureus* is able to “hide” in host cells and therefore could survive standard antibiotic treatment^24^,^25^,^26^. Therefore, it is possible that the asthmatic disease generates the necessary micromilieu that allows *S. aureus* to escape antibiotic eradication. Furthermore, we could show that antibiotic therapy in control children without *S. aureus* colonization in the nasopharynx resulted in lower levels of IL-33 in the NPF than in the corresponding control children who were not treated with antibiotics (Fig. 3d). Previous studies could already show that the immunosuppressant rapamycin (which is chemically related to macrolide antibiotics) could inhibit IL-33 induced airway inflammation^27^. Furthermore, we found that therapy with antibiotics is associated with decreased IL-33 only in control children in the absence of *S. aureus* in nasopharyngeal fluid. However, *IL-33R/ST2* mRNA was found to be downregulated in the control group of children treated with antibiotics as compared to the antibiotic untreated paired group. These data suggest an inhibitory effect of antibiotics on *ST2* mRNA pathway in blood cells and only in the absence of *S. aureus* on IL-33 in the airways of control but not asthmatic children that need further investigations.

IFNβ production in the airways was not increased after antibiotic treatment. Since antibiotic treatment has a huge systemic effect, regulation of mRNA gene expression in blood cells might be better seen than effects of local bacterial colonization. These results suggest a difference in local immune response to antibiotic treatment in children with an atopic airway as compared to non-atopic children.

The micromilieu of the airways in children with asthma might differ significantly from the airway milieu in non-asthmatic children. This provides opportunities in form of biological niches for commensal germs to persist. As it is shown in Fig. 1d, antibiotic treatment in Asthma children was not able to eliminate Gram negative facultative pathogen bacteria of the airways. In contrast to control children, colonization with *H. influenzae* or *M. catarrhalis* was higher after antibiotic treatment in asthmatic children. This might be a hint that bacteria of asthmatic children are either often resistant to common antibiotic treatment with macrolide or cephalosporine antibiotic due to frequent treatment in the individuals, or that an asthmatic airway provides further options for Gram negative bacteria to survive and modify immune response. Further studies in this direction should be performed in a larger population.

It is already known that lipopolysaccharide (LPS), a component of the cell wall of Gram-negative bacteria, exerts a huge variety of immune modulating effects^2^,^28^,^29^. The main pattern recognition receptor (PRR) for LPS is Toll-like receptor 4 (TLR4). High doses of LPS induce a shift towards T helper cell type 1 immune response^30^. By contrast, low doses of LPS induce a shift towards a T helper cell type 2 (Th2) immune response^31^. In our studies, we could show that Gram negative bacteria preferentially induce the release of IFNβ in the NPF of asthmatic children. These children had also induced levels of IL-33 in their airways (Fig. 3a), thus indicating a possible activating effect of IL-33 on circulating ILC2 blunting the inhibiting effect of IFNβ in this group.

ST2, the receptor for IL-33 on T and ILC2 cells, is an important effector molecule of Th2 responses. It negatively regulates type I interleukin 1 receptor (IL-1RI) and TLR4 but not TLR3 signaling by sequestrating the adaptor molecules like MyD88. Inhibition of TLRs by IL-33/ST2 promotes a Th2 response, and also identifies IL-33/ST2 as a key regulator of endotoxin tolerance^32^. TLR3 can therefore activate interferon regulating factor 3 (IRF3) and thus induces IFNβ. The mechanism by which Gram negative respiratory pathogens (*H. influenzae, M. catarrhalis*) induce the elevated IFNβ levels seen in the respective children, is currently unknown.

The contact of bacterial endotoxins like LPS with epithelial cells creates a cellular damage and necrosis^33^. At that point IL-33 is produced by these cells. IL-33 is associated with recurrent wheezing in children with asthma and currently, a polymorphism in IL-33/ST2 has been connected to asthma development in childhood^34^. IL-33 together with thymic stromal lymphopoietin (TSLP) induces inflammation in a Th2 immune response^35^. Thus, IL-33 production induces IL-5 and IL-13 release in asthma, cytokines that are produced by Th2 and ILC2 cells. We could show that asthmatic children were sensitive to antibiotic therapy in terms of down-regulation of IL-33, whereas they do not so in the absence of *S. aureus*. Consistently, IL-33R remained upregulated in asthmatic children in the presence of antibiotic treatment and absence of *S. aureus*. The mechanism of action of antibiotics inhibiting IL-33/IL-33R-ST2 in control, but not in asthmatic children is unknown and needs further investigation. Moreover, we describe here that children with a Gram negative bacterial colonization have increased IFNβ in their NPF. Both these findings could be explained by upregulation of IRF3^36^.

By contrast, when Gram negative bacterial colonization was associated with *S. aureus* and *Streptococcus* species (Gram positive), IFNβ was suppressed thus resulting in elevated ST2 values inspite of the fact that IL-33 was found reduced in this group. This could be explained by immune evasion mechanisms of *S. aureus* and certain *Streptococcus* species^24^,^37^,^38^.

In summary, we have found that IFNβ and IL-33 are associated with Gram negative colonization in the airways of asthmatic children. Moreover, *ST2* mRNA expression in blood was found induced in asthmatic children associated with a mixed Gram- and Gram + bacteria flora in the airways. Furthermore, treatment with antibiotics was found associated with reduced *ST2* mRNA in the blood and IL-33 levels in the airways of control children without *S. aureus* in NPF but not significantly in asthmatic children, although the latter were more often treated with antibiotics.

## Material and Methods

The methods described in this manuscript were carried out in accordance with the approved local and European guidelines.

### Study subjects

Children with or without asthma at the age of four to six years were recruited in the Department of Allergy and Pediatric Pneumology of the Children’s Hospital of Erlangen as part of the Europe-wide study PreDicta (Post-infectious immune reprogramming and its association with persistence and chronicity of respiratory allergic diseases). One aim of PreDicta is to identify altered host-pathogen interactions, molecules and pathways that mediate the establishment and persistence of chronic inflammation in allergic diseases, thus to help to develop new preventive, diagnostic and therapeutic strategies. Therefore, we and the other study centers established and followed up a cohort of preschool children with or without asthma for two years.

Inclusion criteria for cases and controls were (a) the need of a written informed consent from the child’s parents or guardians (the legal custodian must have the verbal, writing and mental ability to understand the intent and character of the study); (b) an age of four to six years at the baseline visit (beginning at the day of the 4th birthday and ending at the day of the 6th birthday); (c) a gestational age of 36 weeks or above; and (d) the diagnosis of asthma within the last two years, confirmed by a pediatrician of the Children’s Hospital in Erlangen. The asthma should be of mild to moderate persistent severity according to the GINA guidelines (2005). There must be three asthma episodes during the preceding 12 months and one of them within the last six months. In addition, the child had to be able to perform at least one peak expiratory flow (PEF) manoeuvre. The controls should not have a history of asthma or wheezing and atopic illness.

Exclusion criteria for the cases of this study were (a) severe or brittle asthma; (b) present immunotherapy or more than six courses of oral steroids during the previous 12 months; (c) other underlying chronic respiratory diseases (e.g., cystic fibrosis, bronchopulmonary dysplasia, immunodeficiencies) except for allergic rhinitis and (d) other chronic diseases except for atopic eczema or chronic medication use.

We recruited 24 asthmatic patients and 21 healthy subjects at our study center in Erlangen. Atopy was proven by at least one positive skin prick test, while asthma was defined in accordance to a physician’s diagnosis of mucus production, bronchial hyperresponsiveness and dyspnoea. The healthy control subjects did not have a history of atopy or asthma. This study was approved by the ethics committee of the Friedrich-Alexander University Erlangen-Nürnberg, Germany (Re-No 4435) and is also registered in the German Clinical Trial Register (Registration number: DRKS00004914). Informed consent was obtained from the parents of all children of the PreDicta study.

### Blinding

Every single participant of the study was assigned a specific number. Only the clinical investigators and study nurses of the Children’s Hospital had access to the full name.

### Nasopharyngeal samples

A nasopharyngeal specimen from control and asthmatic children was collected using a per-nasal applicator swab, which has a tip with flocked soft nylon fiber (E-Swab 482CE, Copan, Italy). Swabs were passed through the nostrils until resistance was felt and they were slowly rotated for five seconds to allow for mucus absorption. In addition, swabs were also rotated against the mucosa of the anterior nares before exiting the nose. The nylon tip was eluted by placing into the E-Swab’s medium.

The nasopharyngeal fluid was divided into aliquots under sterile conditions. A 100 μl aliquot was used for bacterial culture, while the other aliquots were stored at −80 °C until further analysis by ELISA. Within four hours from collection the nasopharyngeal fluid was brought to the Microbiology Institute and used to inoculate two microbiological plates. 50 μl each of the nasopharyngeal fluid were used to inoculate a sheep blood agar and a chocolate agar plate and streaked out using a Drigalski spatula and applying the “triple streak technique”. The plates were incubated at 36 °C in an atmosphere supplemented with 5% CO2 for 48 hours. After 24 hours and 48 hours the plates were evaluated and the bacterial growth was semiquantified (growth in the 1st streak only = few; 1st and 2nd streak = moderate; 1st, 2nd and 3rd streak = numerous). In addition, the following bacterial species were identified: *Streptococcus (S.) pneumoniae, Moraxella (M.) catarrhalis, Haemophilus (H.) influenzae, Staphylococcus (S.) aureus* and *Streptococcus (S.) pyogenes.*

### Antibiotic therapy

With the help of a questionnaire, filled out by the parents after receiving written information from the childrens’ doctors, the number of antibiotic therapies within twelve months prior to the baseline visit and the maximal duration (days) of a particular antibiotic treatment was recorded.

### ELISA

Nasopharyngeal fluids were analyzed for proteins by using ELISA. Human IFNβ was detected by using human ELISA Set Verikine (25–2000 pg/ml, PBL Assay Science, New Jersey, USA). Human IL-33 (23.44–1500 pg/ml) was detected by using a DuoSet sandwich ELISA kit (R&D Systems, Wiesbaden, Germany).

### Isolation of RNA from total blood

Blood samples were collected in Tempus Blood RNA Tubes (Life Technologies, GmbH, Darmstadt, Germany) containing a stabilizing reagent, starting lysis almost immediately after mixing with venous blood, inactivating cellular RNases and precipitating RNA. Isolation of RNA was performed with the MagMAX Kit according to the manufacturer’s protocol (life technologies™, GmbH, Darmstadt, Germany). After, with 1x PBS, the total volume was brought to the desired final volume and probes were centrifuged (15 min, 4 °C, 5000 × g). Crude RNA pellets were washed with Tempus Pre-Digestion Wash and centrifuged again (10 min, 4 °C, 5000 × g). Afterwards RNA was diluted in a mixture consisting of Tempus Resuspension Solution and Tempus Proteinase. Subsequent purification of RNA was performed by using a magnetic bead-based technology. Briefly, binding solution-concentrate, RNA binding beads and isopropanol were added and the samples brought in a magnetic field. Separation of RNA from the solution was automatically performed by the magnetic RNA binding beads. Beads were washed away twice with washing solution 1 and 2 in the magnetic field. After adding elution buffer and using the magnetic field again, supernatant contained the purified RNA.

After isolation, RNA from total blood was reverse transcribed using the first strand cDNA synthesis kit (Thermo Scientific, Darmstadt, Germany).

### Quantitative real-time PCR

Quantitative PCR was used for analyzing mRNA as described for expression of ST2 by using the following set of primers: hST-2 fwd:5′CACGGTCAAGGATGAGCAAG3′ hST-2 rev:5′GCAGAGCAAGTTAGGTTTGCG3′. Hypoxanthine Guanine Phosphoribosyl Transferase (HPRT) served as reference gene and was amplified by using forward (5′-TGA CAC TGG CAA AAC AAT GCA-3′) and reverse (5′-GGT CCT TTT CAC CAG CAA GCT-3′) primers. Reactions contained SYBR^®^ Green (Bio-Rad Laboratories, Munich, Germany), forward and reverse primers, DEPC water and cDNA. Analysis was performed in a CFX96 Real-Time PCR Detection System (Bio-Rad Laboratories, Munich, Germany) with incubation for 2 minutes at 98 °C followed by 50 cycles of 5 seconds at 95 °C and 10 seconds at 60 °C. Afterwards the reaction was stopped with 5 seconds at 65 °C and 5 seconds at 95 °C. The data were analyzed using the 2(−∆∆Ct) method. Primers were purchased from Eurofins-MWG-Operon (Ebersberg, Germany).

### Statistical analysis

The data were evaluated for significant differences by using the one-tailed T test for unpaired data (Graphpad Prism 6, Graphpad Software, Inc., La Jolla, CA, USA). Data are shown as means values ± SEMs. *p < 0.05; **p < 0.01, ***p < 0.001.

## Additional Information

**How to cite this article:** Hentschke, I. *et al*. IL-33/ST2 immune responses to respiratory bacteria in pediatric asthma. *Sci. Rep.* **7**, 43426; doi: 10.1038/srep43426 (2017).

**Publisher's note:** Springer Nature remains neutral with regard to jurisdictional claims in published maps and institutional affiliations.

## Figures and Tables

**Table 1:** Demographic and clinical data of the cohorts of WP1-UK-ER*.

	Control Children	Asthma Children
Number of subjects	21	24
Gender	12 (M) = 57% 9 (F) = 42,8%	15 (M) = 62,5% 9 (F) = 37,5%
Age [years]	4,7 ± 0,18	4,9 ± 0,1
Skin Prick Test positive	25% (2/8)	77% (17/22)^***^
Average age at onset of symptoms [years]	—	2,2
^**^FEV1 < 100%	23,8% (5/21)	54% (13/24)
Allergic Rhinitis	0	33% (8/24)
Atopic eczema	4,76% (1/21)	4% (1/24)
Allergic rhinitis and atopic eczema	0	38% (9/24)
without allergic co-morbidity	95% (20/21)	25% (6/24)
family predisposition to asthma	14% (3/21 maternal)	38% (9/24)
family predisposition to atopy	76% (16/21)	83% (20/24)
both parents with atopy	23,8% (5/21)	46% (11/24)
without family predisposition	23% (5/21)	17% (4/24)

^*^WP1-UK-ER = Work Package 1 within the PreDicta Study at the Universitätsklinikum Erlangen and the University of Erlangen-Nürnberg; **FEV1 = Forced Expiratory Volume in 1 second; ***2 children: skin prick test not done.

**Table 2:** Antibiotic treatment and bacterial nasopharyngeal colonization of the cohorts of WP1-UK-ER.

	Control Children	Asthma Children
>1 antibiotic treatment within last 12 months before recruitment	19% (4/21)	42% (10/24)
≥3 antibiotic treatments within last 12 months before recruitment*	9,5% (2/21)	17% (4/24)
antibiotic treatment in children with *S. aureus*	0% (0/3)	83% (5/6)
antibiotic treatment in children with only *H. influenza and/or M. catarrhalis*	67% (2/3)	100% (5/5)

^*^Most frequently used antibiotics by pediatricians: Control Children: cefaclor, amoxicillin; Asthma Children: clarithromycin, erythromycin, cefpodoxime (justified by the diagnosis: pneumonia).

**Table 3:** Treatment of asthmatic children at the time of recruitment of the cohorts of WP1-UK-ER.

Asthma children	Gram + bacteria: *S. aureus*	Gram- bacteria: *H. influenzae* and *M. catarrhalis*	Number of antibiotic courses within previous 12 months before recruitment	Antibiotic treatment [days]	Antibiotic treatment [component]	Allergic rhinitis	Inhalative treatment	Oral and nasal treatment	Steroid treatment
201	Yes	Yes	3	10	No data	Yes	Fluticasone, Salmeterol 1–0–1	Montelukast 5 mg/d	Yes
202	No	No	1	10	Ciprofloxacin	Yes	Fluticason 1–0–1	Mometason (nasal)	Yes
203	Yes	Yes	1	No data	No data	Yes	Budesonid 0,5 mg 1–0–1	Cetirizin	Yes
204	Yes	No	0	0	—	Yes	Fluticasone, Salmeterol 1–0–1	—	Yes
205	No	Yes	0	0	—	No	Fluticasone, Salmeterol 1–0–1	—	Yes
206	No	No	1	7–10	No data	No	Beclomethasone		Yes
207	No	No	0	0	—	No	Fluticasone, Salmeterol 1–0–1	—	Yes
209	No	Yes	1	7	No data	yes	Budesonide	—	Yes
210	Yes	Yes	2	7	Clarithromycin, Cefpodoxime	yes	Salbutamol	—	No
212	No	No	8	No data	No data	No	Fluticasone, Salmeterol 1–0–1	—	Yes
213	No	No	1	10	No data	No	Fluticasone, Salmeterol 1–0–1	—	Yes
216	No	No	2	No data	No data	Yes	Fluticasone, Salmeterol	—	Yes
217	Yes	Yes	1	7	No data	Yes	Betamethasone 0,1 mg, 1–0–1	—	Yes
223	No	No	2	10	No data	Yes	Budesonide, 2 × 0,2 mg	Montelukast 5 mg	Yes
224	No	No	1	5	No data	Yes	Fluticasone 1–0–1	—	Yes
225	No	Yes	2	10	No data	No	Fluticasone 1–0–1	—	Yes
228	No	No	0	0	—	Yes	Rescue treatment: Salbutamol	—	No
229	No	Yes	1	7–10	Erythromycin, Amoxicillin	Yes	Salbutamol 1x/d	—	No
230	No	No	2	14	No data	Yes	Rescue treatment: Salbutamol	—	No
231	No	Yes	1	4	No data	Yes	Budesonide 0,5 mg, Salbutamol	—	Yes
238	No	Yes	7	7	No data	Yes	Salmeterol, Fluticasone	—	Yes
239	No	Yes	0	0	—	Yes	Rescue treatment	Cetirizin	No
242	Yes	Yes	3	7–10	No data	Yes	Budesonide 0,5 mg	Cetirizin	Yes
243	No	Yes	2	10	Erythromycin	No	Salbutamol, Budesonid 0,5 mg	—	Yes

**Table 4:** Treatment of control children at the time of recruitment of the cohorts of WP1-UK-ER.

Control Children	Gram + bacteria: *S. aureus*	Gram- bacteria: *H. influenzae* and *M. catarrhalis*	Number of antibiotic courses within previous 12 months before B0	Antibiotic treatment [days]	Antibiotic treatment: Component
208	No	No	2	7	No data
211	No	Yes	1	28	amoxicillin + clavulanic acid
214	No	No	0	0	—
215	No	No	0	0	—
218	No	Yes	1	5	No data
219	No	Yes	0	0	—
220	No	No	0	0	—
221	No	Yes	1	7	Cefaclor
222	No	No	0	0	Cefaclor
226	No	No	1	10	—
227	Yes	No	0	0	—
232	Yes	Yes	0	0	—
233	No	No	5	10	phenoxymethylpenicillin, erythromycin, cefaclor
234	No	Yes	0	0	—
235	No	Yes	0	0	—
236	No	Yes	1	10	amoxicillin
237	No	No	4	7	amoxicillin
240	No	Yes	2	7	Cefaclor
241	No	No	1	10	No data
245	Yes	No	0	0	—

**Table 5:** Bacterial nasopharyngeal colonization, antibiotic and asthma medication at the 24 months follow-up visit of the cohorts WP1-UK-ER.

Child number	Asthma (A)/Control (C)	Gram + bacteria: *S. aureus*	Gram- bacteria: *H. influenzae and or M. catarrhalis*	subgrouped in:	Days of antibiotic treatment in the previous 12 months	Component of antibiotic treatment	Asthma medication
214	C	No	Yes	Gr-	7	no data	no medication
215	C	Yes	Yes	Gr−/+	7	no data	no medication
220	C	Yes	No	Gr−/+	0		no medication
221	C	Yes	No	Gr−/+	0		no medication
222	C	Yes	No	Gr−/+	0		no medication
226	C	No	No	PNC	0		no medication
227	C	No	Yes	Gr-	0		no medication
201	A	Yes	Yes	Gr−/+	0		steroid
202	A	No	Yes	Gr−/+	6	no data	steroid
204	A	No	No	PNC	6	Amoxicillin, Cefaclor	no medication
205	A	No	Yes	Gr-	0		no medication
210	A	No	No	PNC	0		no medication
212	A	No	Yes	Gr-	8	no data	steroid
216	A	No	No	PNC	3	no data	steroid
217	A	Yes	No	Gr−/+	10	infectocillin	non-steroid
223	A	No	No	PNC	0		steroid
