# Correction: Corrigendum: IL-33/ST2 immune responses to respiratory bacteria in pediatric asthma

**DOI:** 10.1038/srep46897

**Published:** 2017-08-24

**Authors:** Isabella Hentschke, Anna Graser, Volker O. Melichar, Alexander Kiefer, Theodor Zimmermann, Bettina Kroß, Patricia Haag, Paraskevi Xepapadaki, Nikolaos G. Papadopoulos, Christian Bogdan, Susetta Finotto

**Keywords:** Asthma, Paediatric research, Molecular medicine

10.1038/srep43426

This Article contains an error in the Author Information section, where the following statement was omitted:

“The present work was performed in fulfilment of the requirements for obtaining the degree “Dr. med.” for Isabell Hentschke at the Friedrich-Alexander-Universität (FAU) Erlangen-Nürnberg, Universitätsklinikum Erlangen, Erlangen, Germany”.

